# Dissociation of Hydrofluorocarbon Molecules after Electron Impact in Plasma

**DOI:** 10.1021/acs.jpclett.4c00348

**Published:** 2024-03-19

**Authors:** Dmitry V. Makhov, Gregory Armstrong, Hsiao-Han Chuang, Harin Ambalampitiya, Kateryna Lemishko, Sebastian Mohr, Anna Nelson, Jonathan Tennyson, Dmitrii Shalashilin

**Affiliations:** † School of Chemistry, University of Leeds, Leeds LS2 9JT, United Kingdom; ‡ School of Mathematics, University of Bristol, Fry Building, Woodland Road, Bristol BS8 1UG, United Kingdom; § Quantemol Ltd., 320 City Road, The Angel, London EC1V 2NZ, United Kingdom; ∥ Department of Physics and Astronomy, University College London, London WC1E 6BT, United Kingdom

## Abstract

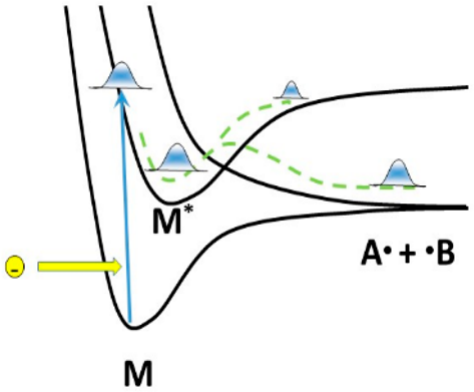

The process of dissociation for two hydrofluorocarbon molecules in low triplet states excited by electron impact in plasma is investigated by *ab initio* molecular dynamics (AIMD). The interest in the dissociation of hydrofluorocarbons in plasma is motivated by their role in plasma etching in microelectronic technologies. Dissociation of triplet states is very fast, and the reaction products can be predicted. In this work, it was found that higher triplet states relax into the lowest triplet state within a few femtoseconds due to nonadiabatic dynamics, such that the simplest *ab initio* MD on the lowest triplet state seems to give a reasonable estimate of the reaction channels branching ratios. We provide evidence of the existence of simple rules for the dissociation of hydrofluorocarbon molecules in triplet states. For molecules with a double bond, the bonds adjacent to the double bond dissociate faster than the other bonds.

Motivated by the search for new environmentally friendly molecules for use in plasma technologies, we study the process of dissociation of hydrofluorocarbon molecules in low-energy triplet states after the electron impact in plasma. Hydrofluorocarbons and other fluoro-organic molecules are used in the microelectronics industry to generate free radicals for plasma etching, one of the main technological processes in microchip production. Many of these molecules and the products of their chemistry in plasma are harmful to the environment. They have a high global warming potential (GWP) and can also cause unwanted reactions in the Earth’s atmosphere.

Breakup patterns of molecules as a result of photodissociation have been well studied theoretically. See for example reviews^1−3^ where references to numerous applications can be found. Similarly, a number of recent studies have focused on developing theories of breakup patterns follow electron impact ionization.^4−7^ This work is aided by extensive data from mass spectroscopy. Fragmentation patterns following electron impact dissociation have been much less well studied. Ziółkowski et al.^8^ used a trajectory hopping approach to study the fragmentation patterns of methane following an R-matrix calculation of electron impact excitation, but there appears to be no work on the fragmentation of larger systems.

Here we investigate the dissociation of two hydrofluorocarbons, 1,1,1,3,3,3-hexafluoropropane (C_3_H_2_F_6_) and 1,3,3,3-tetrafluoropropene (C_3_H_2_F_4_), using *ab initio* molecular dynamics simulations. Both these molecules are of interest because they are considered as a replacement to SF_6_ and C_4_F_8_, traditional components of plasma for plasma etching, and have been investigated experimentally.^9,10^ We use the *ab initio* multiple cloning (AIMC) method,^11,12^ in which an ensemble of Ehrenfest trajectories describes the nuclear motion of the electronically excited molecule. AIMC is a somewhat more rigorous way to treat nonadiabatic transitions than surface hopping employed in ref (8). It has been shown that AIMC can provide an accurate description of nonadiabatic dynamics from first principles.^12−17^ The Quantemol Electron Collisions (QEC) code^18^ that interfaces with the UKRmol+ suite of molecular R-matrix codes^19^ is used to determine the initial triplet states created by electron impact in plasma. Although AIMC was originally developed for the simulation of nonadiabatic dynamics of excited molecules in singlet states in photochemistry, it can equally be used for the dynamics of molecules in low-energy triplet states produced by electron impact. Our study concentrates on these triplet states because they are the lowest electronic states often separated from other excited states by a substantial energy gap.

Our simulations yield the dissociation kinetics of the molecular bonds along with branching ratios for various dissociation channels, which produce neutral free radicals. The branching ratios are important for understanding the chemical composition of plasma but are very difficult to measure experimentally. The calculations appear to yield very simple rules that can be used to predict dissociation channels, even without calculations. Our results show, in particular, that single bonds adjacent to the double bond in C_3_H_2_F_4_ break more efficiently than other bonds.

In the past decade, many quantum and classical molecular dynamics methods have been developed to simulate dynamics of molecules in excited electronic states when electronic excitation energy is transferred into the energy of nuclei, which often results in dissociation. The methods have been implemented in a number of codes^20−22^ and have been applied to a number of photochemical reactions, where the absorption of a visible–UV photon excites the molecule into a singlet state.^1^ The interest in the dynamics of singlet states has been justified by their importance in many processes in photochemistry and photobiology. However, the nature of the initial excitation is not really important, and the same technique can be applied to the molecules excited by electron impact as well, where triplet electronic states are also produced. Indeed, as the lowest excited state is usually a triplet, the dynamics of triplet excitations is the most important in plasma. Following the initial excitation, detailed information about various channels of dissociation after electron impact can be obtained and used in the modeling of chemistry in plasmas. If the cross section σ_*E*_*i*__ of excitation by electron impact of the molecule into a particular electronic state *E*_*i*_ is known, then the cross section of dissociation into a channel *a* can be easily calculated as
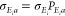
1where *P*_*E*_*i*_*a*_ is the probability of the chemical channel *a* for the molecule excited into electronic state *E*_i_ , which is calculated by molecular dynamics. In its simplest form, *P*_*E*_*i*_*a*_ is a fraction of molecular trajectories ending in the appropriate dissociation channel *a*. In eq 1, both the branching ratio *P*_*E*_*i*_*a*_ and the cross section σ_*E*_*i*__ depend on the temperature, the former through the initial energy of molecules in the dynamics and the latter through the energy of electrons in plasma.

Figure 1 shows the cross section of excitation via electron impact for C_3_H_2_F_6_ and C_3_H_2_F_4_ molecules calculated with the R-matrix method implemented in Quantemol software.^18^ The ground-state configuration of C_3_H_2_F_4_ is (1–20a′, 1–8a″).^2^ The excitation cross sections were calculated at the CASSCF(6e, 6o)/cc-pVDZ level of theory using 19–22a′ and 8–9a″ orbitals in the active space. An R-matrix sphere of radius 10 bohr was used. For C_3_H_2_F_6_, the ground state is (1–13a_1_, 1–7b_1_, 1–11b_2_, 1–6a_2_).^2^ The cross sections were calculated at the CASSCF(8e,8o)/6-311G** level of theory, with an active space consisting of the 13–15a_1_, 6–8b_1_, and 11–12b_2_ orbitals. An R-matrix sphere radius of 10 bohr was found to be sufficient. It can be seen that, while the excitation threshold for the lowest triplet state in C_3_H_2_F_4_ is separated from that for the rest of excited states by a large energy gap of about 5 eV, the two lowest triplet states in C_3_H_2_F_6_ molecule have similar excitation thresholds, and even more triplet states can be excited by electrons with energies just 2 eV higher. Therefore, in principle, many electronic states should be taken into consideration.

**Figure 1:**
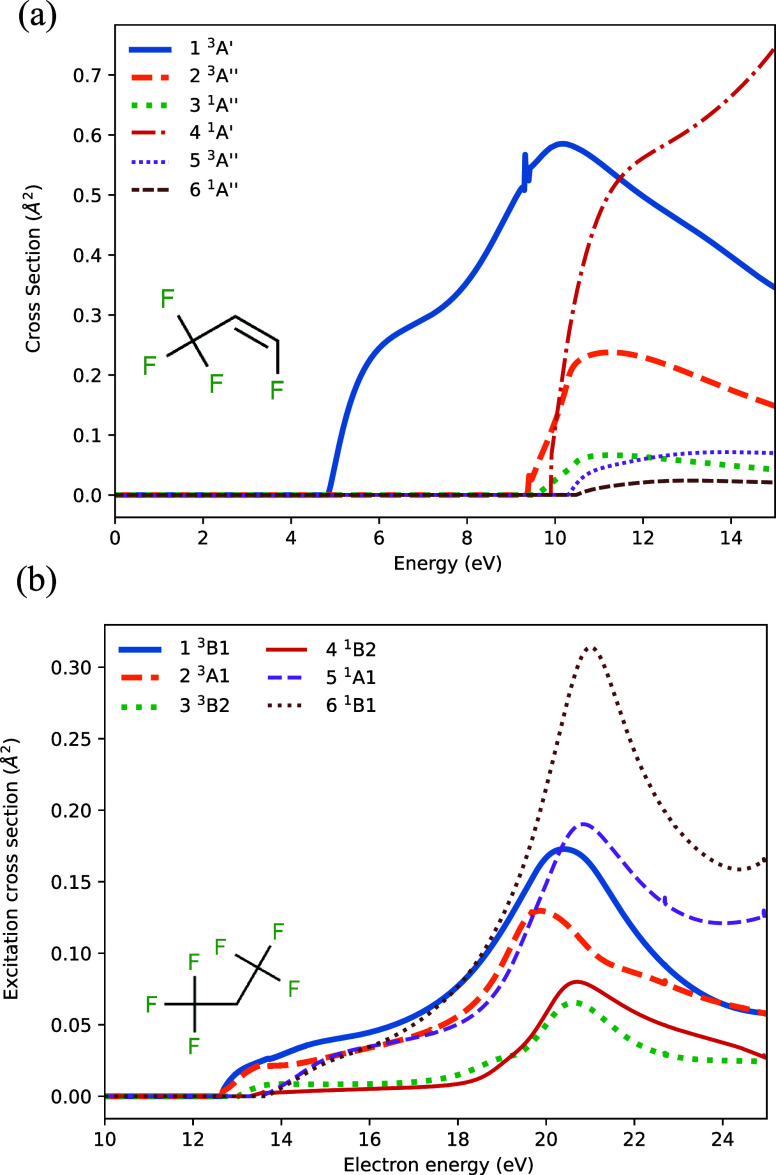
Electron impact excitation cross sections: (A) 1,3,3,3-tetrafluoropropene, where a low triplet state dominates at the electron energies of interest, and (B) 1,1,1,3,3,3-hexafluoropropane, where multiple states contribute for the lowest energies.

We run direct quantum nonadiabatic molecular dynamics simulations using our *ab initio* multiple cloning (AIMC) approach. The dynamics includes several Born–Oppenheimer electronic states with population transfer between them due to nonadiabatic coupling. In the direct dynamics approach, the trajectories of nuclear motion are run without the need for precalculated potential energy surfaces. Instead, the potential energies of the Born–Oppenheimer electronic states, their gradients, and couplings between them are calculated at every time step using electronic structure package (Q-Chem in this case). An ensemble of such trajectories can efficiently simulate the dynamics of an electronically excited molecule.

Our approach is based on Ehrenfest trajectories, which are guided by state-averaged force
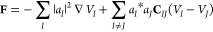
2where *a*_*I*_ are quantum amplitudes for electronic states, *V*_*I*_ are potential energies, and **C**_*IJ*_ is a nonadiabatic coupling vector. The second term here is the so-called Hellmann–Feynman force, which is associated with nonadiabatic electronic population transfer. The quantum amplitudes of electronic states are propagated together with coordinates and momenta of nuclei as
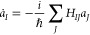
3where *H*_*IJ*_ is the electronic Hamiltonian
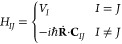
4We do not include spin–orbit coupling here because it is relatively small in hydrofluorocarbon molecules and does not play an important role in their dissociation. Thus, only electronic states with the same spin are considered in our dynamics.

In principle, our AIMC approach can be made fully quantum, as the trajectories here are used just to guide nuclear Gaussian basis functions, and then the time-dependent Schrödinger equation is solved on the basis of trajectory-guided nuclear basis functions (see our reviews^12,23^). We have developed a number of sampling techniques that can be used to describe the initial conditions of the dynamics and the branching of trajectories in the region of nonadiabatic coupling, where the transitions between electronic states occur. The latter is particularly important because Ehrenfest trajectories can become unphysical when several uncoupled electronic states with significantly different gradients have considerable populations. To address this issue, we apply a cloning procedure that results in the branching of trajectories, which reflects the bifurcation of the wave function at conical intersections (see refs (12) and (23) for the details). Each branch gets a weight according to the appropriate Ehrenfest amplitudes. With a sufficient number of coupled basis functions guided by Ehrenfest trajectories, our approach can be converged to the exact fully quantum result.

For the simulation of the dynamics after excitation via electron impact, however, we probably will not need the fully quantum approach with quantum coupling between the trajectories. We will run a number of independent uncoupled trajectories with random initial coordinates and momenta starting from all possible electronic states. Then, we identify the dissociation products and get the statistics for various dissociation channels. The probability *P*_*E*_*i*_*a*_ will then be simply given as the fraction of trajectories that end up in the channel *a* weighted by their branching probabilities, if necessary. If only one electronic state is involved, then AIMC becomes equivalent to standard *ab initio* MD in this case.

The choice of the electronic structure theory method is crucial for the direct dynamics. Previously, in our simulations of photochemistry, we used the CASSCF approach, which unfortunately proved to be unstable for direct dynamics on molecules of interest in plasmas, where the molecules experience strong deformations in the course of dynamics due to high temperatures.

In the direct dynamics approach, the electronic structure is called at every time step of the nuclear dynamics simulation, so the selected electronic structure methods must be cheap and, at the same time, be able to capture most of the dynamic and static correlations during the propagation. After trying several electronic structure methods, we adopted the spin-flip method time-dependent density functional theory (TDDFT)^24^ implemented in the Q-Chem package^25^ for our AIMC direct dynamics. The electronic structure theory used in dynamics is different from that used in the electronic scattering R-matrix calculations. However, as only the two lowest electronic states, which are well separated from other triplets, are considered, we believe that their nature should be the same in both approaches.

It is well-known^26^ that conventional linear-response TDDFT can only deal with single-electron excitation and cannot deal with multiple excitations. Furthermore, it cannot describe sizable hole/electron spatial separation and, also, cannot deal with degeneracies or near-degeneracies between ground and excited states in situations such as dissociations, diradicals, transition states, and conical intersections. The spin-flip TDDFT method covers^27^ most of these TDDFT difficulties by using a high-spin state reference state instead the most stable Kohn–Sham ground state. The spin-flip ansatz applies a spin flipping excitation operator on a single high-spin reference state, generating proper configurations for describing the excited states. This yields accurate potential energy surfaces of the excited states for direct molecular dynamics. However, spin-flip calculations can suffer from spin-contamination due to the incompleteness of the excitation scheme.^24^ This can lead to the artifacts in nonadiabatic coupling terms if two electronic states, in principle with the same spin multiplicity, have different levels of spin contamination. Therefore, it is important to control the expectation value of spin operator ⟨*Ŝ*^2^⟩ during the dynamics.

Our first aim is to estimate the importance of nonadiabatic effects in the process of the triplet state dissociation. We have chosen the C_3_H_2_F_6_ molecule as an example because its two lowest triplet states are very close to each other energetically and have similar excitation cross sections. We run nonadiabatic dynamics for the two lowest triplet states using our AIMC approach with energies and forces provided by Q-Chem^25^ at the spin-flip TDDFT level using the 6-31+G* basis set and BHHLYP hybrid functional.

The initial conditions are generated for *T* = 5000 K using classical Boltzmann distribution for all vibrational modes, with lower temperatures investigated afterward. As the atomic displacements at high temperature would be too large, beyond the applicability of harmonic approximation, we put all thermal energy into the kinetic energy when generating random initial momenta and use the equilibrium geometry as an initial geometry for all trajectories. This should not cause any problems, as the energy redistribution between the kinetic and potential energies happens very fast, within a few vibrational periods. We run 200 initial trajectories starting from both the lowest and second-lowest triplet states, with the number of branches growing in the course of the dynamics as a result of cloning. About half the cloning events happen within first 5 fs of the dynamics, when nonadiabatic transition is the most intensive, and the rest are relatively uniformly spread over the duration of the dynamics. The final number of branches is 492 and 648 for calculations starting from the T_1_ and T_2_ state, respectively.

The control of spin-contamination shows that it is relatively low, especially in the beginning of dynamics: ⟨*Ŝ*^2^⟩ < 2.1 for about 75% of SF-TDDFT calculations in first 10 fs. Although spin-contamination slowly grows over time, this should not significantly affect the results, as the most important nonadiabatic processes occur in the beginning of the dynamics (see below).

Figure 2 shows the average populations of the electronic states as a function of time. One can see very fast nonadiabatic population transfer in the very beginning of the dynamics. The normal-mode analysis shows that this transition is associated primarily with wagging vibrations of the hydrogen atoms. As a result, regardless of the initial state, the population of the lower triplet state became around 75% within just few femtoseconds. This suggests that we can avoid very expensive nonadiabatic calculations and run the dynamics only for the lowest triplet state. In order to test this suggestion, we run the dynamics for the molecule in the lowest triplet state without nonadiabatic effects taken into account and compare the results with those produced by AIMC calculations. One can see from Figure 3 that there is little difference between the dissociation kinetics produced by this simple molecular dynamics on a single lowest triplet state potential energy surface and the AIMC dynamics, even when the latter starts from the upper triplet state. Some difference is found only for C–H bond dissociation, where AIMC dynamics give slightly higher dissociation yield; this can be explained by extremely fast C–H bond dissociation, which happens even faster than the molecule relaxation to the lower state.

**Figure 2:**
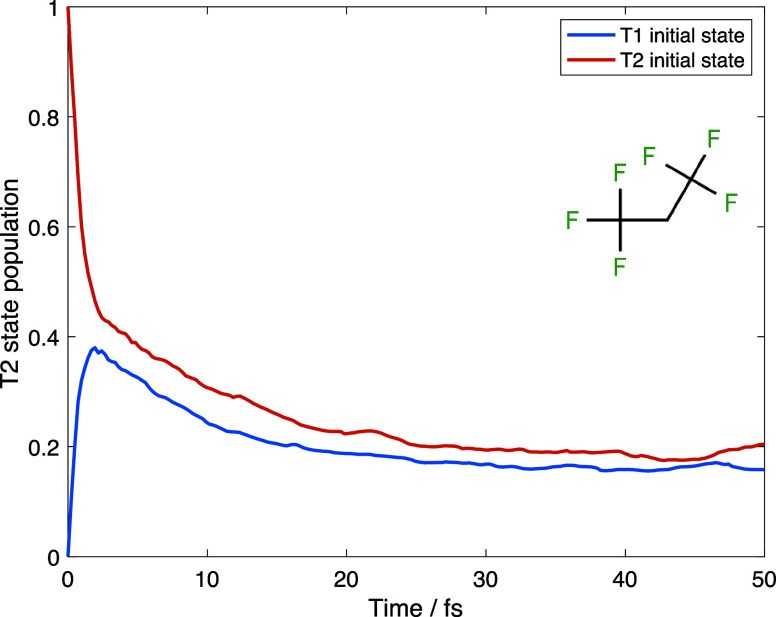
Average populations of the upper triplet state of C_3_H_2_F_6_ molecule as a function of time given by AIMC nonadiabatic dynamics calculations that use lower (blue) and upper (red) triplet states as the initial state.

**Figure 3:**
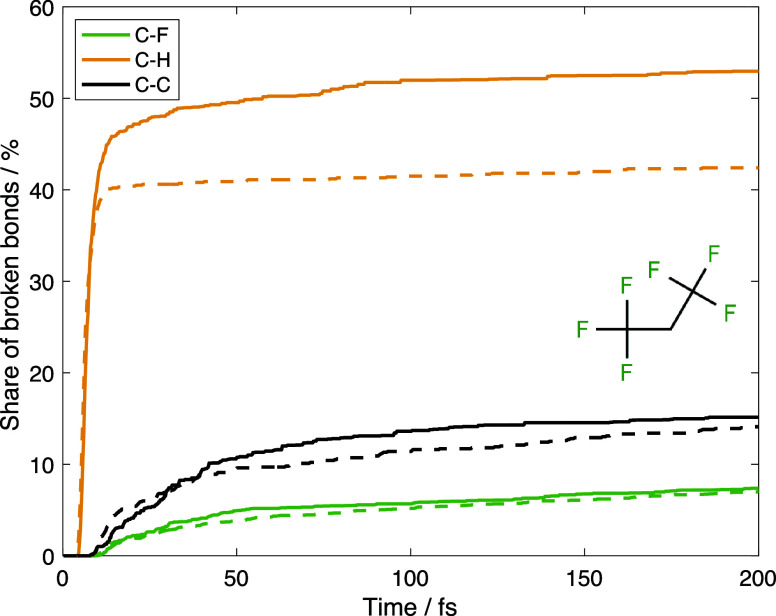
Comparison of dissociation kinetics of a C_3_H_2_F_6_ molecule in a triplet state given by AIMC nonadiabatic dynamics simulations with initial state T2 (solid) and by the molecular dynamics on T1 potential energy surface (dashed). The number of broken bonds was a function of time.

Thus, the simulation of the dissociation in plasma in some cases does not require the use of the expensive nonadiabatic dynamics, and the essential details of the process can be captured by running the dynamics on the lowest triplet potential energy surface (either because only one state is initially excited, as in the case of C_3_H_2_F_4_, or because the molecule quickly relaxes to the lowest triplet state, as in the case of C_3_H_2_F_6_). The rapid relaxation of triplet excitation was also found for methane,^8^ where the same conclusion about the importance of the lowest triplet state was made when computing fragmentation patterns. Surely, more calculations for other molecules are needed before any general conclusions can be made.

Considering the above, we run extensive calculations for C_3_H_2_F_6_ dissociation using cheaper single potential energy surface dynamics that include only the lowest triplet state. We study the dissociation kinetics at 5000, 3000, 2000, and 1000 K; for each temperature, we run an ensemble of 500 trajectories for 600 fs. The bond is considered broken when its length exceeds the 5 Å threshold.

Figure 4 shows the dissociation kinetics for all types of bonds at different temperatures. About half of the C–H bonds break quickly, suggesting that electronic excitation is located at these bonds. The dissociation yield for the C–H bond practically does not depend on temperature, which is consistent with the barrierless dissociation. Then, the gradual excitation transfer leads to the breaking of C–C bonds, followed by slow C–F bond breaking.

**Figure 4:**
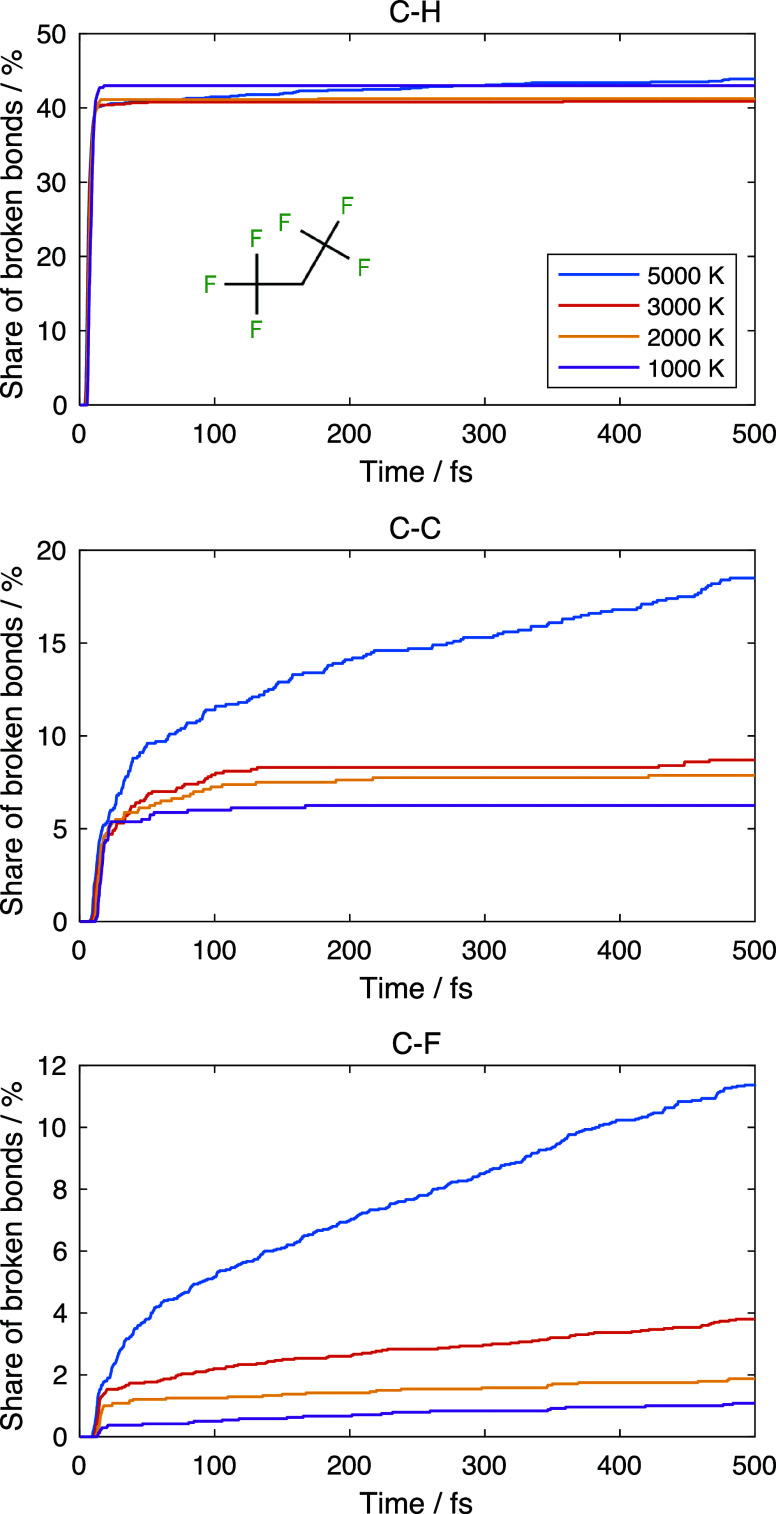
Number of C–H (upper), C–C (middle), and C–F (lower) bonds broken as a function of time for different temperatures for a C_3_H_2_F_6_ molecule.

The character of C–C bond breaking kinetics is somewhat between that for C–H and C–F bonds. About 40% of C–H bonds break within the first 5 fs, and then the dissociation essentially stops. For C–C bonds, about 5% of them break quickly but later than C–H bonds; then, the dissociation gradually continues, at least at higher temperatures. Unlike the C–H bonds, the dissociation of C–F bonds mostly happens gradually, and only a minor fraction of them breaks fast within the first 10–20 fs of the dynamics.

Table I lists the identified fragments for the C_3_H_2_F_6_ molecule at the end of the dynamics at different temperatures. The results are given for 500 molecules. One can see that there are no undissociated molecules, even at 1000 K. The dominating small radicals are H, F, CF_2_, and CF_3_. The large number of C_3_HF_6_ radicals, which is nearly equal to the number of H radicals at 1000 and 2000 K, indicates that the dissociation of a C–H bond essentially eliminates the probability of any other dissociation at lower temperatures. The large number of CF_2_, CF_3_, and C_2_H_2_F_3_ radicals at all temperatures shows that a significant number of F radicals are produced by the dissociation of the C–F bond in CF_3_ radicals that were produced by earlier C–C bond dissociation.

**Table I:** Number of Fragments for C_3_H_2_F_6_ Dissociation per 500 Molecules after 600 fs of the Dynamics at Different Temperatures

	5000 K	3000 K	2000 K	1000 K
H	410	407	407	424
F	308	125	70	40
C_3_HF_6_	195	370	400	420
C_3_HF_5_	112	31	6	1
CF_2_	104	51	41	26
CF_3_	62	33	42	43
C_2_H_2_F_3_	33	56	61	63
CF	29	7	1	1
HF	28	2	3	
C_2_HF_3_	28	5	2	1
C_2_H_2_F_2_	17	17	6	2
CH_2_	15	4	6	1
C_3_F_5_	15			
C_2_HF_2_	14	2	1	
CHF	11			
CH_2_F	11	1	1	1
C_2_HF_4_	7	1		
C_3_H_2_F_5_	7	12	16	10
C_3_HF_4_	6		1	
C_3_F_4_	5			
C_2_HF	4			
C_3_H_2_F_4_	4	1		
H_2_	3			
C_2_F_2_	3			
C_2_F_3_	3			
C_3_F_6_	3			1
C	1			
CF_4_	1			
CH	1			
C_2_F_4_	1			
C_2_H_2_	1			
C_2_H_2_F	1			

Figure 5 shows the dissociation kinetics for C_3_H_2_F_4_ at 5000 K. The bond that breaks most efficiently here is the single C–C bond: about 40% of them is broken within 600 fs.

**Figure 5:**
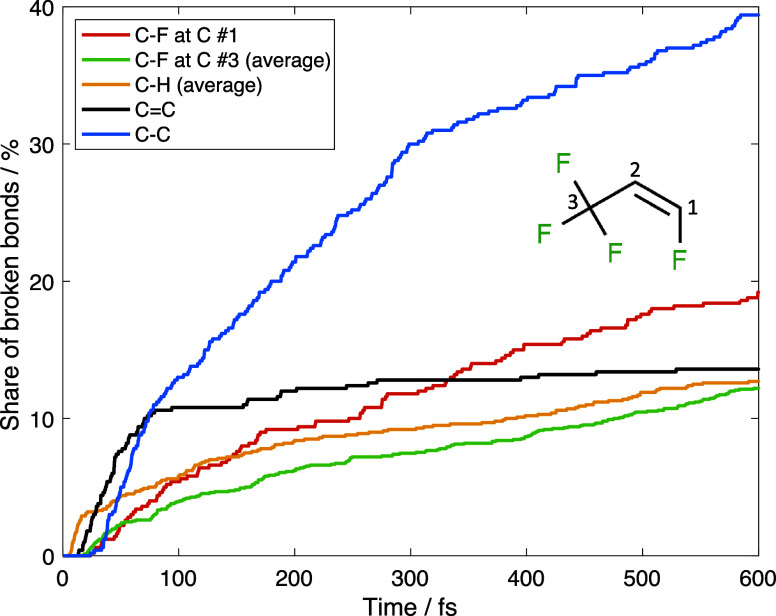
Dissociation kinetics for the C_3_H_2_F_4_ molecule at 5000 K.

The character of C–H bond breaking kinetics in the C_3_H_2_F_4_ molecule is very different from the C_3_H_2_F_6_ case: C–H bonds here break gradually with approximately the same rate as C–F bonds. On the other hand, the kinetics of double C=C bond breaking resembles that for the C–H bond in the C_3_H_2_F_6_ molecule: the bonds break very intensively in the first 30 fs of the dynamics, and then the process essentially stops. This agrees with the suggestion that the electronic excitation is initially localized at the double bond: the lowest triplet state results from the removal of an electron from a bonding π-orbital and adding an electron to a π*-antibonding orbital. This state is isolated from all other excited states by a 5 eV energy gap. Therefore, *ab initio* MD calculations on this single state are well justified even without additional simulations of nonadiabatic dynamics.

Comparing the dissociation rate for different C–F bonds, we have found that the bond at the C atom involved in double bonding breaks more efficiently than C–F bonds at the other C atom. This can be explained by the initial localization of the excitation at the double C=C bond, which then gradually spreads to the neighboring bonds. This observation is extremely important, as it can help to establish simple rules that can be used to predict dissociation channels even without calculations. More detailed calculations for C_2_H_2_F_4_ molecule will be a part of our next work.

In summary, we performed simulations of the dissociation of 1,1,1,3,3,3-hexafluoropropane (C_3_H_2_F_6_) and 1,3,3,3-tetrafluoropropene (C_3_H_2_F_4_) molecules in their lowest excited triplet states generated by electron impact. We run *ab initio* nonadiabatic molecular dynamics with potential energy surfaces and forces calculated “on the fly” by the electronic structure code. The nonadiabatic dynamics calculations are expensive. We demonstrate that, at least for the molecules under consideration, accurate dissociation kinetics can be obtained by running much cheaper molecular dynamics calculations involving only the lowest triplet state. This approach can work even in the case when multiple triplet states are excited initially, e.g., for C_3_H_2_F_6_ dissociation, because the molecule ends up in the lowest triplet state within few femtoseconds.

Running the lowest triplet state dynamics on a large number of CPUs, we were able to propagate a large number of trajectories and accumulate good statistics for C_3_H_2_F_6_. We have studied in detail the kinetics of dissociation for C–H, C–C, and C–F bonds at different temperatures and identified various dissociation channels.

Calculations for C_3_H_2_F_4_ demonstrate that the double bonds play an important role, as the triplet excitation is initially localized there. The excitation then moves toward the neighboring single bonds, which break more efficiently than the bonds further away from the double bond. The most abundant products detected are produced by breaking of single bonds adjacent to double bonds. In our next work, we will attempt to simulate dissociation patterns of molecules that have been studied experimentally. The goal of these simulations will be to understand the rules, which should enable us to predict products of dissociation and, therefore, enable control of the chemical composition of plasma. Our effort to find simple rules of dissociation of molecules after electron impact supports that of recent works,^28,29^ where dissociation of molecules in low temperature plasma have been investigated experimentally.

*Ab initio* molecular dynamics is computationally expensive even when only one triplet state is included. At each time step. an electronic structure package is used to calculate potential energy surface and its derivatives, which determine forces between the molecules. A much faster approach would be to use analytical force fields similar to those used in molecular dynamics of biomolecules.^30^ Force fields for molecules in triplet states obviously differ from those of molecules in their ground electronic state. However, by accumulating more *ab initio* MD data, we will try to generate force fields for triplet excited states. Recently, an attempt has been made to model triplet state force field with machine learning parametrization,^31^ but other parametrizations, similar in spirit to those used for the force fields of ground state,^30^ may be possible.

Hydrofluorocarbons and other organofluorine molecules are broadly used in the microelectronics industry for various plasma technologies. Recently they came under scrutiny due to their potential environmental damage, and the importance of finding new less environmentally damaging molecules has recently been highlighted.^28,29^ We demonstrate that simulations of the dynamics of their dissociation are possible and can be done in a manner similar to how simulations of photodissociation were done previously. Also, perhaps, simple rules that allow predictions of dissociation channels can be found, improving plasma modeling for applications.

Previously, simulations of dynamics involving excited electronic states were mostly focused on photochemical processes, where singlet states are produced via photon absorption. In this Letter, we are expanding to the dynamics after excitation by electron impact, which involves low-energy triplet states that are energetically separated from higher-lying singlet states. However, the methodology is very much the same for the triplet and singlet states. Simulating the dissociation of the molecules in triplet states excited by electron impact is actually simpler than that for photodissociation because the potential energy surface of the triplet state generally includes repulsive parts. To the best of our knowledge, theoretical simulations of dissociation of molecules in triplet states are very rare. One example is the work in ref (8), where dissociation of methane has been investigated using surface hoping method. Given the practical importance of triplet state dissociation for plasma technologies, we plan to continue this work and want to bring this problem to the attention of the molecular dynamics community.
